# The interplay between IGF-1R signaling and Hippo-YAP in breast cancer stem cells

**DOI:** 10.1186/s12964-023-01088-2

**Published:** 2023-04-20

**Authors:** Yu-Tzu Chan, Ruey-Jen Lin, Ya-Hui Wang, Tsai-Hsien Hung, Yenlin Huang, John Yu, Jyh-Cherng Yu, Alice L. Yu

**Affiliations:** 1 Institute of Stem Cell and Translational Cancer Research, Chang Gung Memorial Hospital at Linkou, Taoyuan, Taiwan; 2 Chang Gung University, Taoyuan, Taiwan; 3 Department of Anatomic Pathology, Chang Gung Memorial Hospital at Linkou, Taoyuan, Taiwan; 4 Department of Surgery, Tri-Service General Hospital, Taipei, Taiwan; 5 School of Medicine, National Tsing-Hua University, Hsinchu, Taiwan; 6 Department of Pediatrics, University of California in San Diego, San Diego, CA USA; 7 Genomics Research Center, Academia Sinica, Taipei, Taiwan

**Keywords:** Hippo, YAP, IGF-1, IGF-1R, Breast cancer stem cells

## Abstract

**Background:**

Both IGF-1R/PI3K/AKT/mTOR and Hippo pathways are crucial for breast cancer stem cells (BCSCs). However, their interplay remains unclear.

**Methods:**

Four triple negative breast cancer cell lines derived from CSC of two patient-derived xenografts (PDXs), AS-B145, AS-B145-1R, AS-B244, and AS-B244-1R, were used to elucidate the role of YAP in BCSCs. YAP silenced BCSCs were analyzed by cell proliferation, aldehyde dehydrogenase (ALDH) activity, mammosphere formation, and tumorigenesis. The effects of modulating IGF-1R and IGF-1 on YAP expression and localization were evaluated. The clinical correlation of YAP and IGF-1R signaling with the overall survival (OS) of 7830 breast cancer patients was analyzed by KM plotter.

**Results:**

Knockdown of YAP abates the viability and stemness of BCSCs in vitro and tumorigenicity in vivo. Depletion of IGF-1R by shRNA or specific inhibitor decreases YAP expression. In contrast, IGF-1 addition upregulates YAP and enhances its nuclear localization. YAP overexpression increased the mRNA level of IGF-1, but not IGF-1R. Data mining of clinical breast cancer specimens revealed that basal-like breast cancer patients with higher level of IGF-1 and YAP exhibit significantly shorter OS.

**Conclusions:**

YAP contributes to stemness features of breast cancer in vitro and in vivo. The expression and localization of YAP was regulated by IGF-1R and YAP expression in turns upregulates IGF-1, but not IGF-1R. Clinically, higher level of YAP and IGF-1 significantly correlated with shorter OS in basal-like breast cancer. Taken together, these findings suggest the clinical relevance of interplay between YAP and IGF-1/IGF-1R pathway in sustaining the properties of BCSCs.

Video Abstract

**Supplementary Information:**

The online version contains supplementary material available at 10.1186/s12964-023-01088-2.

## Background

Cancer stem cells (CSCs) represent a small population of cancer cells with the capacities of self-renewal and differentiation [1]. For breast cancer, cells expressing CD24^-/low^/CD44^+^ [2] or high ALDH activity [3] are reported to be enriched in CSC population. CSC plays an important role in metastasis and resistance to chemo- and radiotherapy, which are relevant to the clinical outcome and therapeutic relapse [4]. Many signaling pathways have been shown to be crucial for regulation of BCSCs, such as PI3K/AKT/mTOR, Wnt, Hedgehog, Notch, JAK-STAT, and NF-κB [5].

Recently, Hippo pathway has also been implicated. Hippo pathway consists of a core kinase cascade in which MST1/2 phosphorylates LATS1/2 kinase. Once LATS1/2 is activated by phosphorylation, the downstream effectors, YAP and TAZ, are continuously phosphorylated by LATS1/2, resulting in the inhibition of their activity as transcription cofactors. Knockdown of TAZ alone inhibits mammosphere formation ability in MDA-MB-231 breast cancer cells [6]. YAP cooperates with beta-catenin to regulate CSCs-related traits in a Wnt/Met double mutant mouse model, which developed tumors with human basal-like breast cancer characteristics [7].

Previously, we showed IGF-1R/PI3K/AKT/mTOR pathway to be crucial for BCSC properties [8]. Since IGF-1R and YAP were both upregulated in sorafenib-resistant hepatocellular carcinoma (HCC) [9], we investigated the interplay between IGF-1R and Hippo-YAP pathway in BCSCs. In this study, we used breast cancer PDX models to show that IGF-1R signaling regulates YAP expression and its localization. On the other hand, YAP overexpression upregulated IGF-1 expression, but not IGF-1R. Clinically, higher expression of both YAP and IGF-1, but not IGF-1R, contribute to poor outcome of patients (“Materials and Methods” see Additional file 2).

## Results

### YAP expression in BCSCs contributes to enhanced cell proliferation, stemness features, and tumorigenicity

**Fig. 1:**
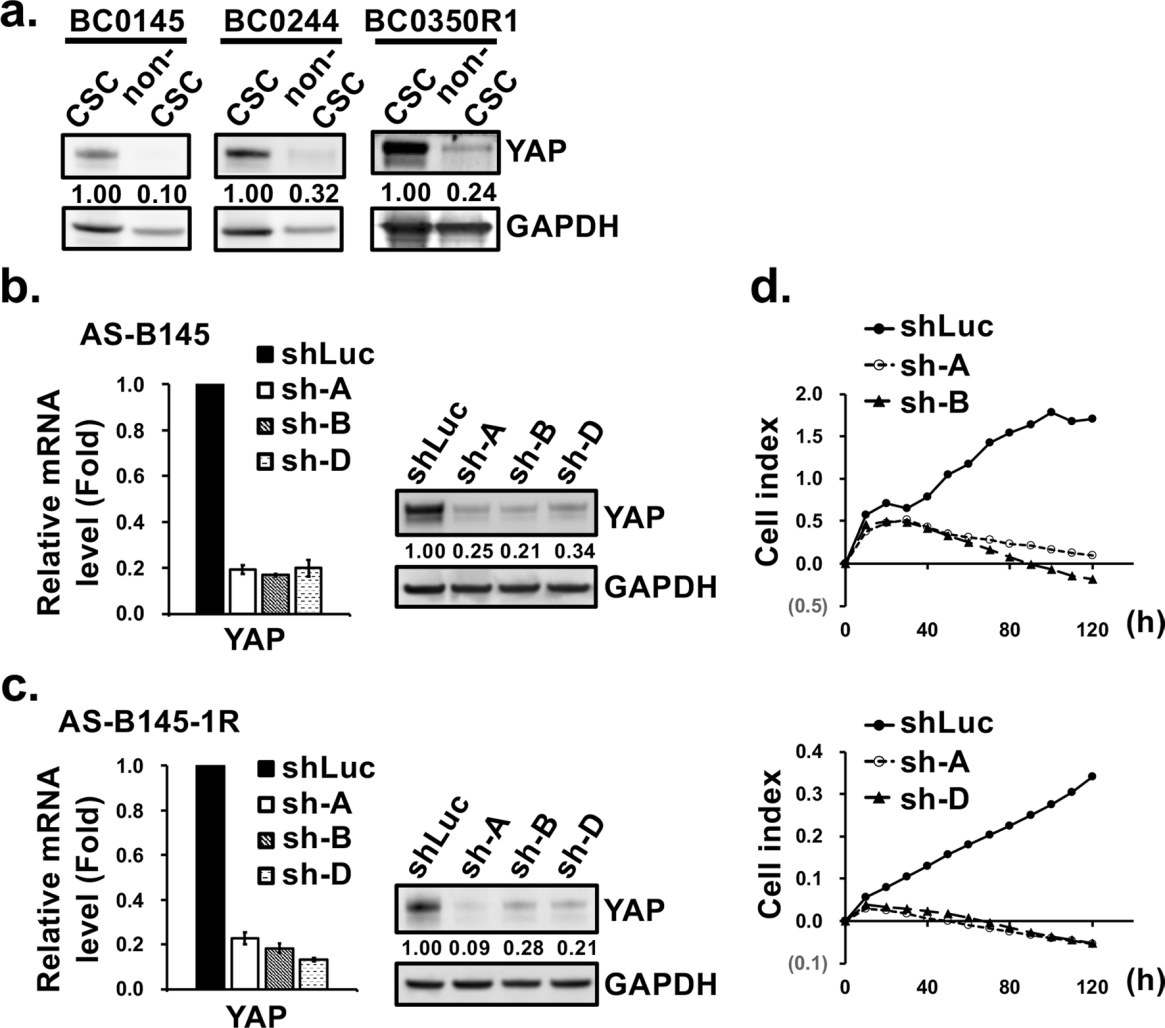
Higher expression of YAP in BCSCs and its silencing reduces cell proliferation **a** The protein expression of YAP in BCSCs sorted from xenografts of BC0145 (H2k^d−^CD24^−^CD44^+^), BC0244 (H2k^d−^ALDH^+^), and BC0350-R1 (H2k^d−^ALDH^+^) were compared with non-BCSCs. GAPDH protein served as the internal control for normalization. YAP expression in BCSC was set as 1.0 for comparison to non-BCSCs. **b** AS-B145 cells were infected with lentiviral vector containing shRNAs for YAP (shYAP) or shLuc control. The total RNA and proteins were harvested 3 d after infection for RT-qPCR and western blotting, respectively. The normalized YAP expression of shLuc cells was set as 1.0 for comparison to values of shYAP infected cells (sh-A, sh-B, and sh-D). **c** The expression of YAP mRNA and protein were determined in shRNAs infected AS-B145-1R cells. **d** The growth curves of shRNA infected AS-B145 (upper panel: shLuc, sh-A, and sh-B) and AS-B145-1R (lower panel: shLuc, sh-A, and sh-D) cells were determined using the xCELLigence system over a period of 120 h. Sh-A: shYAP clone A; sh-B: shYAP clone B; sh-D: shYAP clone D

**Fig. 2:**
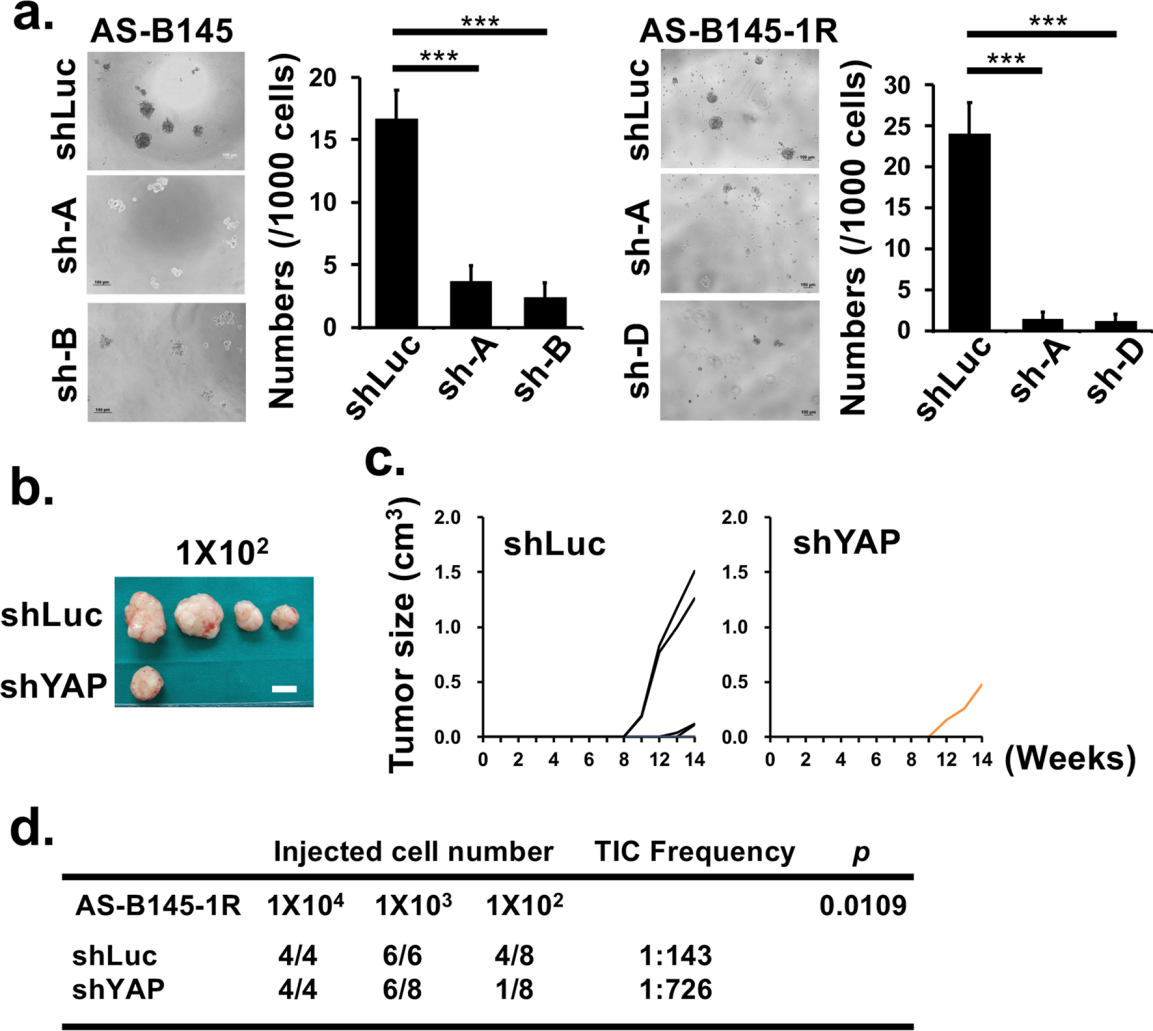
YAP expression contributes to enhanced stemness features and tumorigenesis in BCSCs. **a** shRNAs infected AS-B145 (left panel) and AS-B145-1R (right panel) cells were cultured for mammosphere formation for 7 days and the number of spheres were counted (1000 cells/well in a 96-well plate format). **b** and **c** 1 × 10^2^ of shRNAs infected AS-B145-1R cells were injected into mammary fat pad of NSG female mice and tumor sizes were monitored weekly. The photographs of tumors **b** and tumor growth curves **c** were recorded. The white line represents 1 cm. **d** Serial dilutions of YAP silenced cells (shYAP) and control cells (shLuc) from 10^2^ to 10^4^ cells were injected into NSG mice. The CSC frequency was calculated by ELDA software. *** *P* < 0.001 as compared with the control group (shLuc) using the *t*-test

Previously, we had established three PDXs of human breast cancer, including BC0145, BC0244, and BC0350R1 in mice and identified H2K^d−^CD24^−^CD44^+^ in BC0145 and ALDH^+^ cells in BC0244 and BC0350R1 as markers for their CSCs [8, 10]. Subsequently, our comparative phosphoproteomic analysis revealed greater phosphorylated YAP at Serine 61 (2.7 and 19.1 folds) and Threonine 63 (2.8 and 18.7 folds) in BCSCs than in non-BCSCs in two repeated experiments [11] (Additional file 1: Fig. S1). This was consistent with greater expression of YAP in BCSC than non-BCSC of these three PDXs as shown in western blot analysis (Fig. 1a). Next, the effects of YAP silencing on cell proliferation and mammosphere formation ability were assessed in two stem-like cell lines, AS-B145 and AS-B145-1R [12], which were derived from CD24^−^CD44^+^ and CD221^+^ cells, respectively, of BC0145. Transduction of AS-B145 cells by three shRNA clones (sh-A, -B, and -D) reduced the mRNA levels of YAP to ~ 20% of control (Fig. 1b, left panel) and the protein levels to 25%, 21%, and 34%, respectively, of control (Fig. 1b, right panel). Similarly, YAP in AS-B145-1R cells was effectively repressed by shRNAs at mRNA and protein level (Fig. 1c). Using xCELLigence system, sh-A and sh-B infected AS-B145 cells showed a lower cell index than controls, suggesting that YAP depletion impeded cell growth (Fig. 1d, upper panel). Similar results were obtained in AS-B145-1R cells infected by shYAP (clones A and D) (Fig. 1d, lower panel). YAP silencing of AS-B145 cells also diminished the mammosphere-forming capacity from 16.7 ± 2.3 in shLuc control to 3.7 ± 1.2 and 2.3 ± 1.2 in sh-A and -B, respectively (*P* < 0.001, Fig. 2a). Similar findings were observed in AS-B145-1R, with the reduction of mammospheres from 24 ± 3.8 in shLuc to 1.3 ± 0.9 and 1.2 ± 0.9 in sh-A and sh-D, respectively (*P* < 0.001). Another PDX-derived cell lines, AS-B244 and AS-B244-1R, which were sorted from BC0244 by ALDH^+^ and CD221^+^, respectively, were used to confirm the YAP functions. As shown in Additional file 1: Fig. S2a, transduction of AS-B244 cells by two shRNA clones (sh-A and -D) reduced the mRNA levels of YAP to ~ 50% and ~ 60% of control, respectively. YAP silencing of AS-B244 cells decreased the ALDH activity from ~ 41% in shLuc control to ~ 20% and ~ 29% in sh-A and -D, respectively. In addition, YAP depletion in AS-B244-1R cells reduced the mammosphere-forming capacity from 9.7 ± 3.5 in shLuc control to 0.2 ± 0.4 in sh-A (Additional file 1: Fig. 2b), indicating that YAP is important for stemness features in BCSCs. To determine the contribution of YAP to tumorigenesis in vivo, NSG mice were injected with serial dilutions of YAP silenced cells (shYAP) and control cells (shLuc) from 10^2^ to 10^4^ cells. As expected, YAP silenced AS-B145-1R cells displayed lower engraftment capacity with smaller tumor size than controls, especially in the groups injected with 1 × 10^2^ cells (Fig. 2b and c). Using ELDA software [13], the tumor forming-frequency for shLuc control cells (1 in 1.43 × 10^2^) was 5.08-fold of shYAP cells (1 in 7.26 × 10^2^), indicating that YAP downregulation significantly dampened tumorigenicity in vivo (Fig. 2d).

### IGF-1R regulates the expression and localization of YAP in BCSCs

**Fig. 3:**
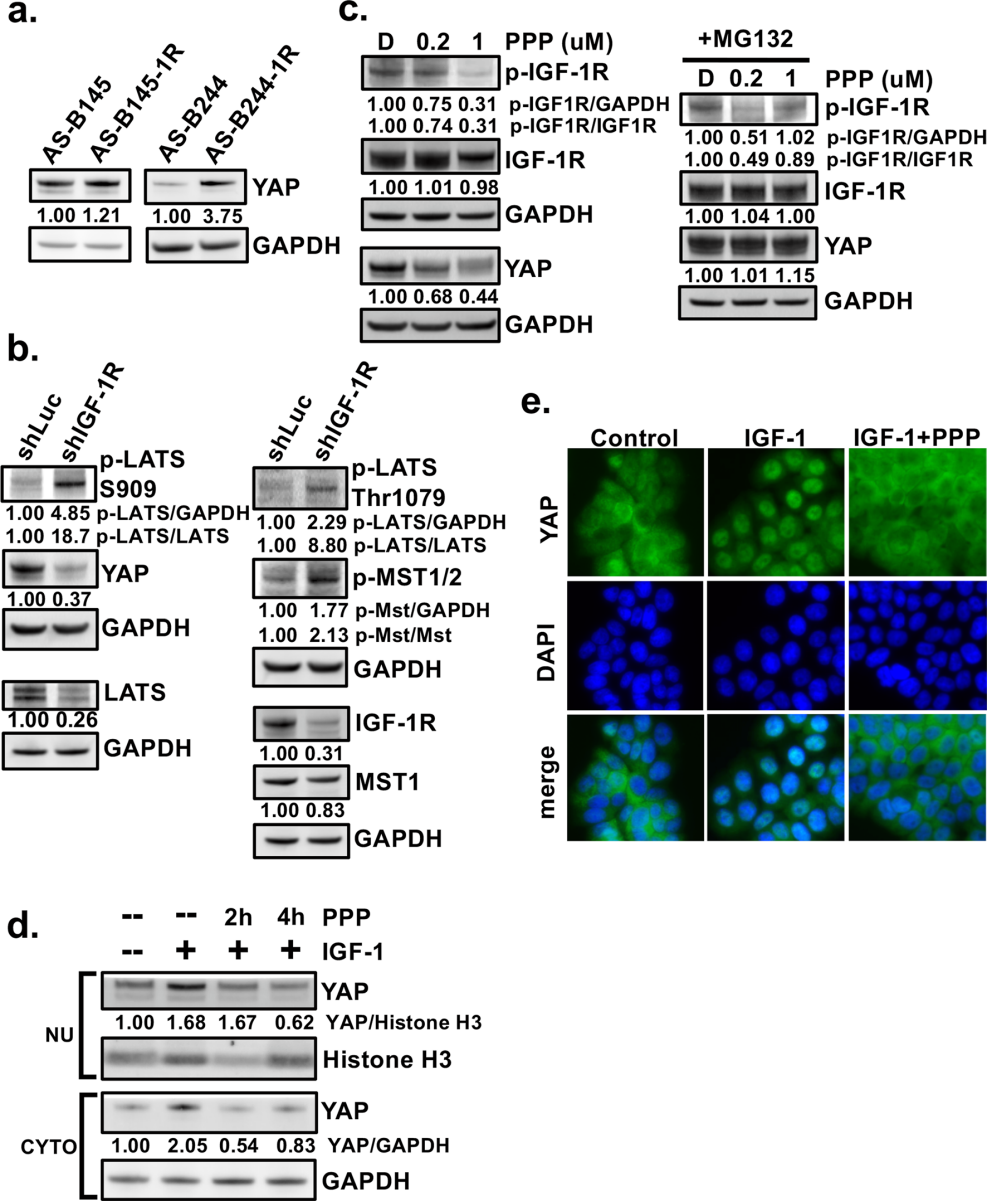
IGF-1R signaling regulates the expression and subcellular localization of YAP. **a** The protein expression of YAP in AS-B145 versus AS-B145-1R and AS-B244 vs. AS-B244-1R was determined by western blotting. The expression levels of YAP in AS-B145 or AS-B244 were set as 1.0 for comparison to values of their IGF-1R enriched subclones. **b** The expression of Hippo kinases and YAP was determined by western blotting in IGF-1R silenced AS-B145-1R cells. **c** Left: Twenty-four hours after PPP treatment (0.2 and 1 μM), p-IGF-1R, IGF-1R, and YAP were determined in AS-B145-1R cells. Right: AS-B145-1R cells were treated with PPP and MG132 simultaneously for 24 h. The protein expressions were determined by western blotting. **d** After incubation with IGF-1 (20 ng/mL) for 30 min, AS-B145-1R cells were treated with PPP (1 μM) for 2 h or 4 h. The proteins were extracted from cytoplasm and nucleus for determination of YAP expression by western blotting.** e** Immunofluorescence staining of YAP (green) and DAPI (blue) in AS-B145-1R cells treated for 30 min with IGF-1 (20 ng/mL) alone or in combination with the PPP (1 μM) for 4 h

As shown in Fig. 3a, the expression levels of YAP in AS-B145-1R were found to be higher than AS-B145. Similar results were obtained by comparing AS-B244-1R with AS-B244. These findings suggested a correlation between the expression of YAP and IGF-1R. Transduction of AS-B145-1R cells with shIGF-1R led to upregulation of core components of Hippo pathway with increase of p-MST1/2 to 2.13-fold of control cells (Fig. 3b). The phosphorylation of LATS was also higher in shIGF-1R cells than control (18.7-fold and 8.8-fold increases at Ser909 and Thr1079, respectively). In contrast, the expression of YAP decreased to 37% of control in IGF-1R silenced AS-B145-1R cells. In line with this, treatment of AS-B145-1R (Fig. 3c, left panel) and AS-B244-1R cells (Additional file 1: Fig. S3) with specific IGF-1R inhibitor PPP at 1 μM reduced the expression of YAP to 44% or 42% of control, respectively. Moreover, downregulation of YAP was rescued by MG132, a specific proteasome inhibitor, indicating that IGF-1R modulates YAP degradation (Fig. 3c, right panel). Along the same line, activation of IGF-1R signaling by IGF-1 increased YAP expression in AS-B145-1R cells (Additional file 1: Fig. S4). Since IGF-1R signaling could increase the nuclear translocation of YAP [9], we evaluated the impacts of IGF-1 on the subcellular localization of YAP. Incubation of AS-B145-1R cells with IGF-1 increased YAP levels in nuclear and cytoplasmic compartments, both of which were reduced by addition of PPP (Fig. 3d). Furthermore, using immunofluorescence staining, nuclear accumulation of YAP was clearly discernible upon IGF-1 treatment, but diminished by subsequent addition of PPP (Fig. 3e). These findings indicate that YAP expression and localization was regulated by IGF-1R signaling.

### The correlation of YAP with IGF-1 in basal-like breast cancer is important for cancer progression

**Fig. 4:**
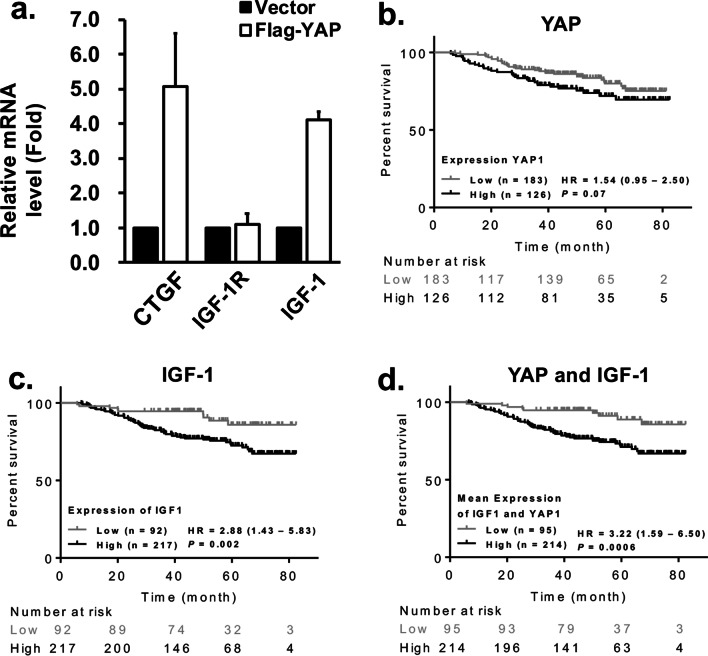
Clinical relevance of the expression of IGF-1R, IGF-1, and YAP in basal-like breast cancer patients. **a** RT-qPCR analysis of CTGF, IGF-1R, and IGF-1 mRNA expression in YAP overexpressing cells. The expression of mRNA was presented as fold relative to shLuc. **b** The clinical relevance of overall survival and IGF-1 or YAP expression in 309 basal-like breast cancer patients were analyzed by KM plotter software. Gene expressions of **b** YAP, **c** IGF-1, and **d** mean expression level of YAP and IGF-1were bisected into high and low expression group using the best cut-off value in KM plotter. Mean expression of IGF-1 and YAP was calculated by KM plotter. HR, hazard ratio

To explore the interplay between YAP and IGF-1R signaling, the mRNA level of IGF-1R and IGF-1 was evaluated in YAP overexpressing cells by RT-qPCR. As shown in Fig. 4a, the expression of a known YAP downstream target, CTGF [14], is increased by 5.1 ± 1.5 fold. The mRNA level of IGF-1 was upregulated by 4.1 ± 0.2 fold, but not IGF-1R (1.1 ± 0.3 increase of control), suggesting that YAP may regulate the expression of IGF-1, but not IGF-1R. To decipher the clinical relevance of YAP, IGF-1R and IGF-1, data mining by KM plotter of patients with basal-like breast cancer was evaluated. As shown in Additional file 1: Fig. S5 and Fig. 4b, patients with higher expression of IGF-1R [Hazard Ratio: 1.38, CI 95% (0.85–2.23),* P* = 0.19] (Additional file 1: Fig. S5) or YAP [Hazard Ratio: 1.54, CI 95% (0.95–2.50), *P* = 0.07] (Fig. 4b) showed a trend for shorter OS, although they did not reach statistical significance. On the other hand, up-regulation of IGF-1 [Hazard Ratio: 2.88, CI 95% (1.43–5.83),* P* = 0.002] was associated with short OS significantly (Fig. 4c). Furthermore, higher level of combination of IGF-1 and YAP exhibits even more significantly shorter OS [Hazard Ratio:3.22, CI 95% (1.59–6.50), *P* = 0.0006] (Fig. 4d). Taken together, these clinical findings supported the interplay between YAP and IGF-1/IGF-1R pathway in tumor progression.

## Discussion

Several lines of evidence have indicated that dysregulation of Hippo-YAP pathway contributes to the tumorigenesis in various human cancers [15]. The interaction of serum response factor (SRF) with YAP mediated the expression of numerous mammary stem cell signature genes to induce the mammary stem cell-like properties in basal-like breast cancer [16]. The regulation of YAP by IGF-1R signaling was reported in diffuse large B-cell lymphoma (DLBCL) [17] and sorafenib-resistant HCC [9]. IGF-1R signaling promoted cell growth by activation of FAK and YAP in TNBC cells [18]. These findings are consistent with our demonstration of interplay between YAP and IGF-1R signaling, contributing to CSC properties in TNBC PDX-derived CSC lines (Additional file 1: Fig. S6). Depletion of IGF-1R by shRNA or specific inhibitor decreased YAP expression. In contrast, IGF-1 addition upregulated YAP and enhanced its nuclear localization. Additional file 3 provides uncropped western blots for Figs. 1, 3, and Figs. S2–S4 in Additional file 1

Although YAP upregulated the expression of IGF-1R in sorafenib-resistant HCC [9], in our study, the expression of IGF-1R was not significantly increased in YAP overexpressing cells. Our finding was corroborated by the results of data mining that TNBC patients with higher expression of IGF-1R did not show significantly shorter OS. This is consistent with another study showing no correlation between IGF-1R expression and OS in TNBC patients [18]. On the other hand, IGF-1 was upregulated in YAP expressing cells and its high expression level conferred adverse impact on the clinical outcome. Although patients with higher expression of YAP was not significantly correlated with shorter OS, the combination of YAP and IGF-1 exhibited significantly shorter OS in TNBC. Previously, up-regulation of IGF-2 was observed in YAP overexpressed medulloblastomas [19]. However, there was no direct evidence supporting regulation of IGF-1 or IGF-2 by YAP, which awaits future studies.

## Supplementary Information

**Additional file 1**. Supplementary Figures

**Additional file 2**. Materials and Methods

**Additional file 3**. Uncropped western blots for Figs. 1, 3, and Figs. S2–4 in Additional file 1

## Data Availability

The datasets used in this study are available from the corresponding author on reasonable request.

## Abbreviations

BCSCs: Breast cancer stem cells
OS: Overall survival
ALDH: Aldehyde dehydrogenase
HCC: Hepatocellular carcinoma
PDX: Patient-derived xenograft
SRF: Serum response factor
DLBCL: Diffuse large B-cell lymphoma
TNBC: Triple-negative breast cancer
