# Supplementary material for: The interplay between IGF-1R signaling and Hippo-YAP in breast cancer stem cells
Source: Cell Commun Signal. 2023 Apr 20;21:81. doi: 10.1186/s12964-023-01088-2 (PMC10120239; doi:10.1186/s12964-023-01088-2)

**Supplementary Information**

**Additional file 1: Fig. S1-S6**

**Supplementary Fig. 1 Higher expression of p-YAP in BCSCs than non-BCSCs.**

Relative fold expression of phosphorylated YAP at Serine 61 and Threonine 63 was determined in BCSCs and non-BCSCs of BC0145 PDX tumor in two independent experiments. The expression of p-YAP in non-BCSCs was set as 1.0 for comparison to values of p-YAP in BCSCs.

**Supplementary Fig. 2 YAP is important for stemness features in BCSCs.**

a. Left panel: AS-B244 cells were infected with lentiviral vector containing shRNAs for YAP (shYAP) or shLuc control. The total RNA were harvested 3 d after infection for RT-qPCR. The normalized YAP expression of shLuc cells was set as 1.0 for comparison to values of shYAP infected cells (sh-A and sh-D). Right panel: BCSCs subpopulation enriched in shRNA infected AS-B244 cells were determined by ALDEFLOUR assay. b. Left panel: The expression of YAP protein was determined in shRNAs infected AS-B244-1R cells. Right panel: Mammosphere formation of shRNAs infected AS-B244-1R cells was determined after culture for 7 days (1000 cells/well in a 96-well plate format).

**Supplementary Fig. 3 Inhibition of IGF-1R reduces YAP expression.**

The effects of PPP treatment on Hippo pathway, including p-LATS, LATS, YAP, and CTGF, were examined by western blotting in AS-B244-1R cells. At 24 h after PPP treatment (1 μM), cells were harvested for protein extraction.

**Supplementary Fig. 4 Addition of IGF-1 increases YAP expression.**

AS-B145-1R cells were treated with IGF-1 (20ng/ml) over the 30 to 120 min time period. Western blotting was performed to determine the expression of p-IGF-1R, IGF-1R, p-AKT, AKT, p-LATS, LATS, and YAP.

**Supplementary Fig. 5 The clinical relevance of YAP and IGF-1R.**

The clinical relevance of overall survival and IGF-1R expression in 309 basal-like breast cancer patients were analyzed by KM plotter software.

**Supplementary Fig. 6 Histopathology of human primary tumor, PDX, and PDX-derived cells.**

Tumor of BC0244 patient, BC0244 PDX, AS-B145-1R, and MCF-7 were collected and fixed with 4% formalin and paraffin embedded. AS-B244-1R, AS-B145, and SKOV3 cells were collected and formed cell blocks. Five μm sections were cut and mounted on microscope slides. Both of tumors and cell blocks sections were stained with ER, PR, and Her2. Sections were examined by pathologists. MCF7 as positive control of ER and PR staining. SKOV3 as positive control of Her2 staining.

**Supplementary Fig. 1**


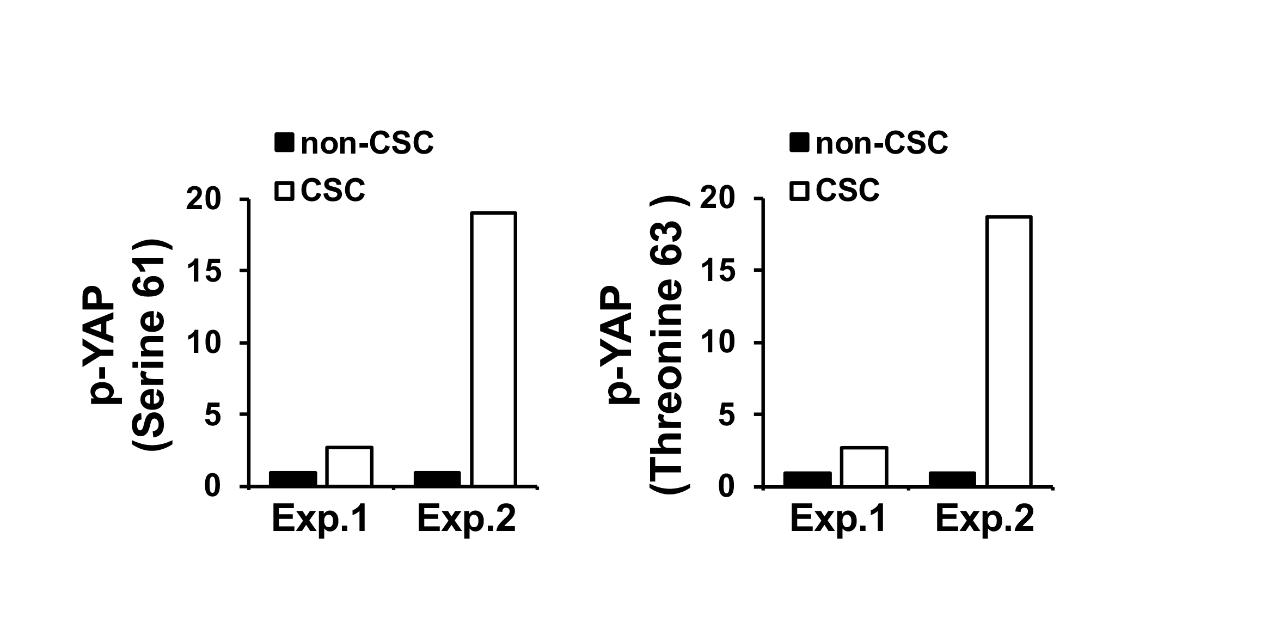


**Supplementary Fig. 2**

**Supplementary Fig. 3**

**Supplementary Fig. 4**

**Supplementary Fig. 5**


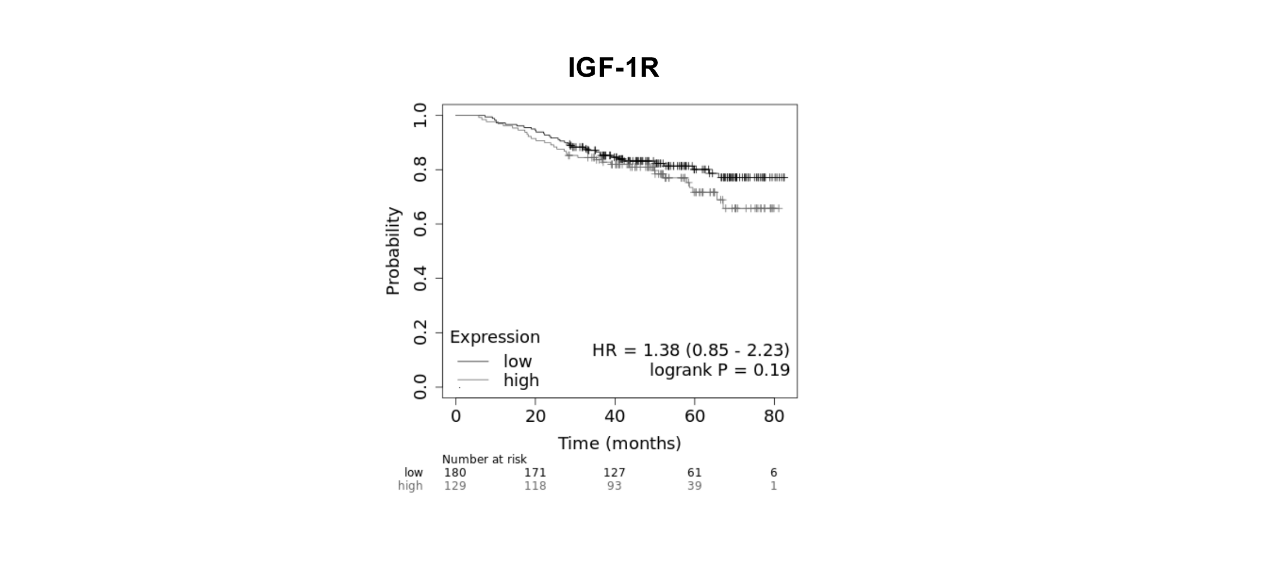


**Supplementary Fig. 6**


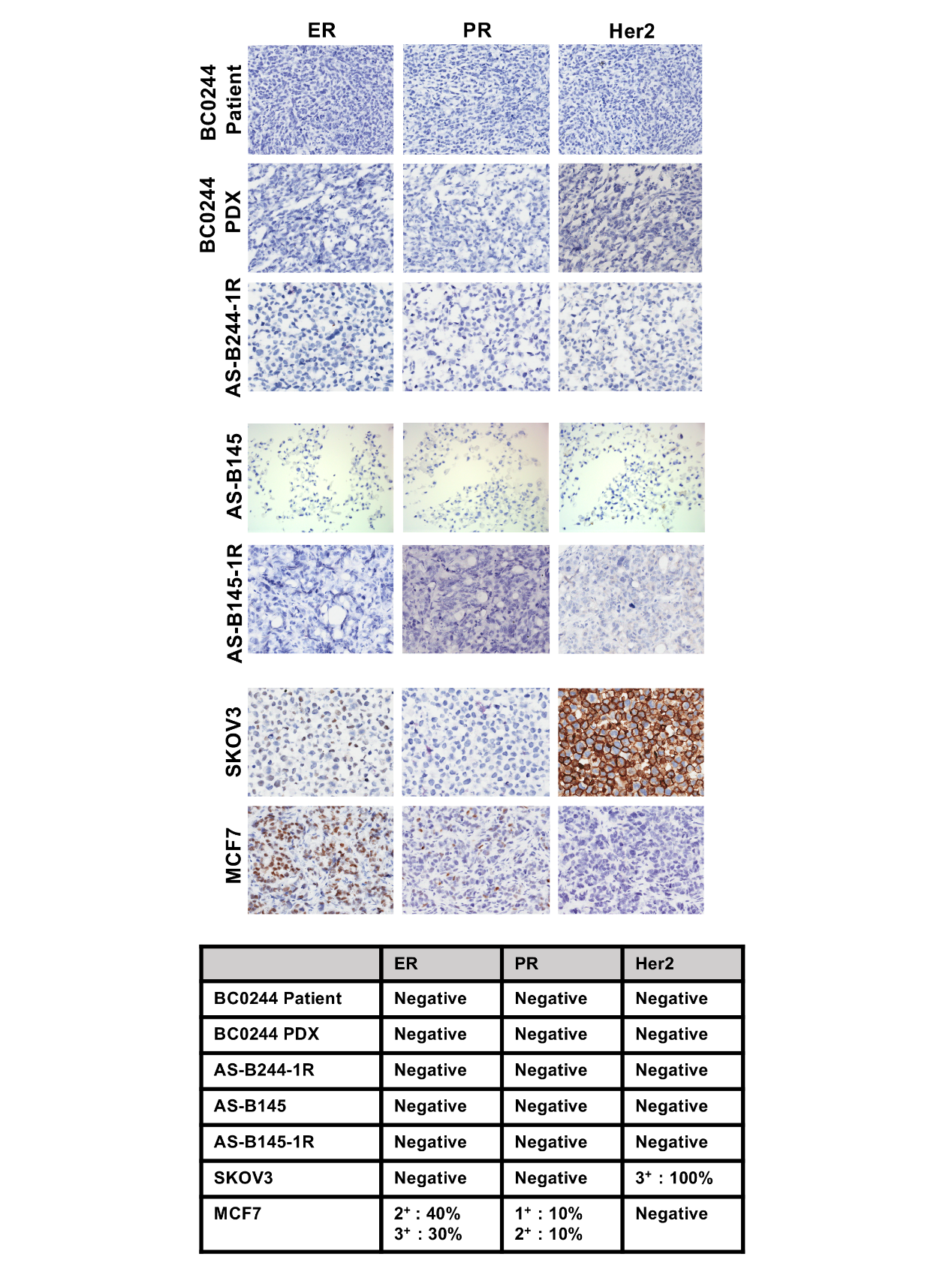

Supplement: Supplementary file 2 — Additional file 1. Supplementary Figures [file 12964_2023_1088_MOESM2_ESM.docx]
