# Supplementary material for: The interplay between IGF-1R signaling and Hippo-YAP in breast cancer stem cells
Source: Cell Commun Signal. 2023 Apr 20;21:81. doi: 10.1186/s12964-023-01088-2 (PMC10120239; doi:10.1186/s12964-023-01088-2)
Supplement: Supplementary file 3 — Additional file 2. Materials and Methods [file 12964_2023_1088_MOESM3_ESM.docx]

**Supplementary Information**

**Additional file 2: Supplementary Materials**

**Cell culture**

293T and MDA-MB231 cells were maintained in DMEM supplemented with 10% (v/v) fetal bovine serum. AS-B145 and AS-B244 cells were derived from H2K^d–^CD24^–^CD44^+^ BCSCs of the BC0145 and H2K^d–^ALDH^+^ BCSCs of the BC0244 xenograft, respectively. AS-B145-1R, and AS-B244-1R cells were derived from CD221^+^ of BC0145 and BC0244, respectively. All of PDX-derived CSC lines were cultured in modified Eagle's medium, 10% (v/v) fetal bovine serum, 10 μg/ml insulin at 37 °C with 5% CO_2_.

**Antibodies and reagents**

MG132 (#474790), picropodophyllin (PPP, IGF-1R inhibitor, #407247), and Akt IV (AKT inhibitor, #124011) were purchased from Calbiochem. Anti-YAP antibodies were from Epitomics (#2060-1) and Cell Signaling Technology (#8418). Anti-p-IGF-1R (Tyr1165/1166, #sc-101704) and anti-Histone H3 (#sc-8654) were from SantaCruz Biotechnology. Anti-IGF-1R (#3027), Anti-p-LATS (Ser909, #9157) , anti-p-LATS (Thr1079, #8654), anti-LATS (#3477), anti-p-MST1(Thr183)/MST2 (Thr180) (#3681), and anti-MST1 (#3682) antibodies were from Cell signaling Technology. Anti-GAPDH (#100118) was from GeneTex Inc.

**RT-qPCR**

Total RNA was extracted from cells using TRIzol reagent (#15596-026, Invitrogen). Briefly, total RNA was reverse transcribed into cDNA using TaqMan Reverse Transcription Reagents (#N8080234, Applied Biosystems). Real-time PCR was performed using FastStart SYBR Green Master (#04673484001, Roche Applied Science). Amplification was done with the 7300 Sequence Detection System (Applied Biosystems). 10 ng of cDNA and specific primers were used for qPCR amplification. The threshold cycle (Ct) values were determined using the default threshold settings. The GAPDH gene was used as an internal control for normalization. The following primers were used: YAP, forward primer 5’-TGTCCCAGATGAACGTCACAGC-3’, reverse primer 5’-TGGTGGCTGTTTCACTGGAGCA-3’; GAPDH, forward primer 5’-CTGCTCCTCCTGTTCGACAGT-3’, reverse primer 5’-ACCTTCCCCATGGTGTCTGA-3’; CTGF, forward primer 5’-CTTGCGAAGCTGACCTGGAAGA-3’, reverse primer 5’-CCGTCGGTACATACTCCACAGA-3’; IGF-1, forward primer 5’-CTCTTCAGTTCGTGTGTGGAGAC-3’, reverse primer 5’-CAGCCTCCTTAGATCACAGCTC-3’.

**Western blotting**

Cells were harvested and lysed by RIPA buffer with phosphatase and protease inhibitors. Proteins were separated by electrophoresis on 4 to 12 % gradient NuPAGE (Invitrogen) and transferred to PVDF membrane (Millipore). Membranes were incubated with blocking buffer containing 5% BSA for 1 hour at room temperature and then incubated with respective primary antibodies overnight at 4 degree. Subsequently, membranes were washed and incubated with appropriate secondary antibodies (Promega) for 2 h. The membranes were probed with ECF system. Fluorescent signals were scanned using a Typhoon9400 Variable Mode Imager (Amersham BioScience)

**Knockdown of YAP expression**

shRNAs were purchased from the National RNAi Core Facility of the Institute of Molecular Biology/Genomics Research Center, Academia Sinica, Taiwan. The following shRNAs were used: shYAP-A (TRCN0000107266): CCGGGCCACCAAGCTAGATAAAGAACTCGAGTTCTTTATCTAGCTTGGTGGCTTTTTG; shYAP-B (TRCN0000107267): CCGGCAGGTGATACTATCAACCAAACTCGAGTTTGGTTGATAGTATCACCTGTTTTTG; shYAP-D (TRCN0000107268): CCGGGACCAATAGCTCAGATCCTTTCTCGAGAAAGGATCTGAGCTATTGGTCTTTTTG. The shRNA plasmid against luciferase served as a negative control (shLuc). The procedure for lentiviruses production and infection into cells was described previously [1, 2].

**Gene expression data analysis**

Gene expression data and survival information of the 7830 breast cancer patients were collected in the KM plotter, which was established based on the 55 microarrays datasets from Gene Expression Omnibus (GEO) [3]. The significance of overall survival and gene expression level were calculated by Kaplan-Meier method and log-rank t test in KM plotter program.

**Mammosphere formation assay**

shRNA infected cells were plated in 96 well ultralow attachment plates with a density of 2,000 cells per well (Corning). Cells were grown in serum-free DMEM/F12 supplemented with 0.4% BSA, B27 (1:50, Invitrogen), 20 ng/ml EGF and 20 ng/ml bFGF (BD Biosciences), and 10 ng/ml insulin (Sigma). The mammospheres were cultured for 7–10 d. The diameter >100 μm of mammospheres were counted.

**Aldefluor assay**

To measure the ALDH activity of shRNA infected cells, the Aldefluor assay was performed according to manufacturer’s (Stemcell Technologies) guidelines. Briefly, cells were suspended in Aldefluor assay buffer and stained with ALDH subtract, Bodipyaminoacetaldehyde (BAAA), for 30 minutes at 37 °C. A fraction of cells was incubated under identical condition in the presence of the ALDH inhibitor, diethylamino benzaldehyde (DEAB). After incubation, cells were analyzed by flow cytometer.

**Tumorigenicity in vivo**

AS-B145-1R cells were infected with lentiviral shRNAs for 3 days. shYAP clone #A was used for YAP knockdown. 1x10^4^, 1x10^3^, or 1x10^2^ of the viable puromycin-resistant AS-B145-1R cells were mixed with matrigel for subcutaneous injection into the mammary fat pads of NSG mice. Tumor formation was monitored weekly after inoculation.

**Cell proliferation assay**

The cell proliferation was performed using the xCELLigence system and presented as cell index. Briefly, shRNA infected cells (5,000 cells/well) were plated in E-plate 96-well (Agilent), and the cell index was continuously monitored for 5 days.

**Immunofluorescence Assay**

Cells were plated on coverslips in 12-well plates. After treatment, cells were fixed with 4% paraformaldehyde for 20 min and permeabilized for 5 min at RT with PBS 0.1% Triton X-100. Primary antibody (anti-YAP, 1:50 dilution) was diluted in 10% BSA containing blocking buffer for overnight staining. The Alexa-488 conjugated secondary antibody (Thermo Fisher Scientific) was stained for 1.5 h at room temperature. DAPI was then used for DNA staining.

**Statistical Analysis**

The results of mammosphere formation were analyzed by two-tailed t-tests. Survival analyses were performed using the Kaplan-Meier method, and the log-rank test was used to identify significant differences. All statistical analyses were performed using GraphPad Prism version 6.0 statistical software. *P* < 0.05 was considered as significant.

**References**

1. Chang WW, Lee CH, Lee P, Lin J, Hsu CW, Hung JT, Lin JJ, Yu JC, Shao LE, Yu J *et al*: **Expression of Globo H and SSEA3 in breast cancer stem cells and the involvement of fucosyl transferases 1 and 2 in Globo H synthesis**. *Proc Natl Acad Sci U S A* 2008, **105**(33):11667-11672.

2. Chan YT, Lai AC, Lin RJ, Wang YH, Wang YT, Chang WW, Wu HY, Lin YJ, Chang WY, Wu JC *et al*: **GPER-induced signaling is essential for the survival of breast cancer stem cells**. *Int J Cancer* 2020, **146**(6):1674-1685.

3. Gyorffy B: **Survival analysis across the entire transcriptome identifies biomarkers with the highest prognostic power in breast cancer**. *Comput Struct Biotechnol J* 2021, **19**:4101-4109.
