# Supplementary figures and images for: The interplay between IGF-1R signaling and Hippo-YAP in breast cancer stem cells
Source: Cell Commun Signal. 2023 Apr 20;21:81. doi: 10.1186/s12964-023-01088-2 (PMC10120239; doi:10.1186/s12964-023-01088-2)

Figure 1

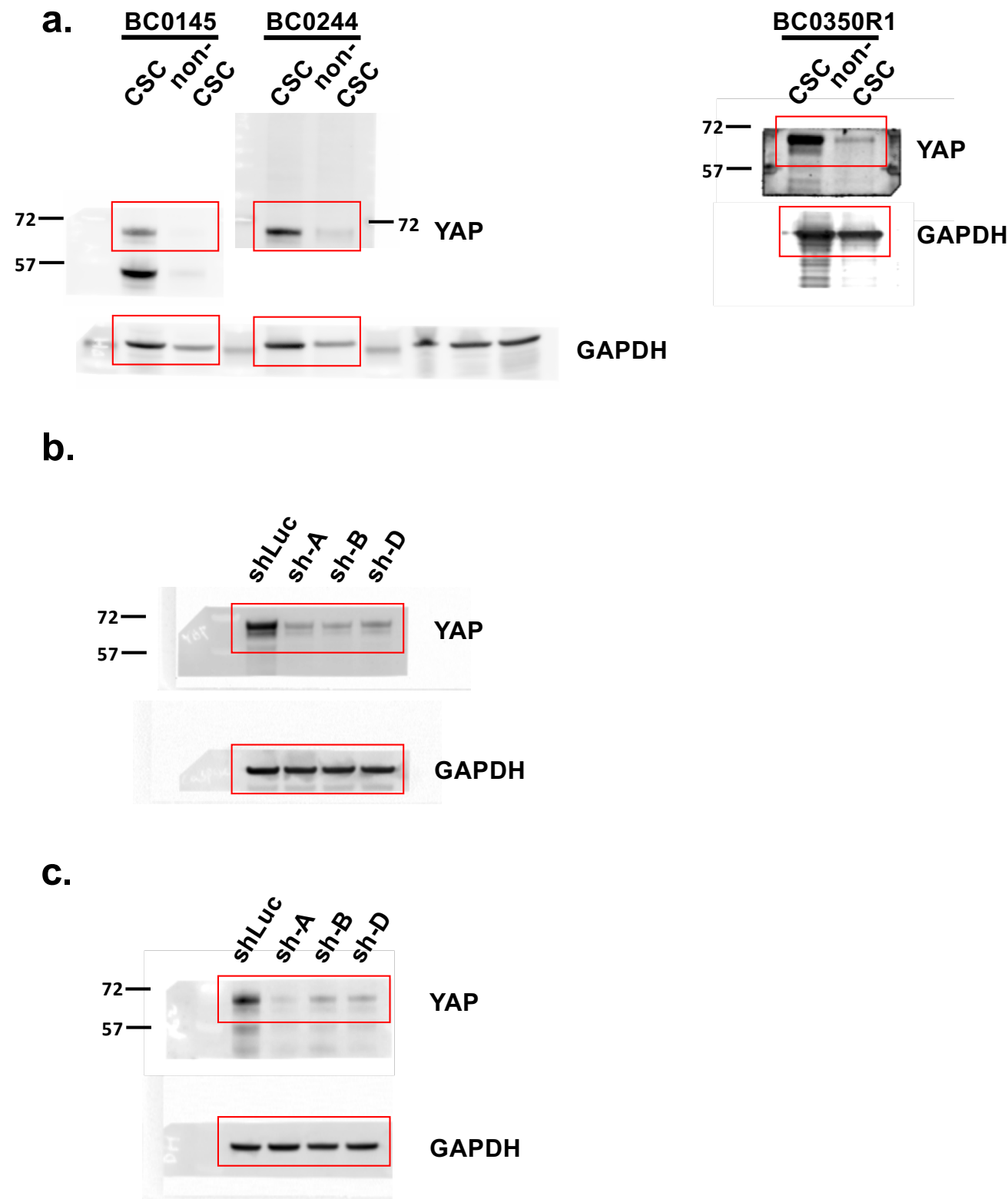

Figure 3

a.

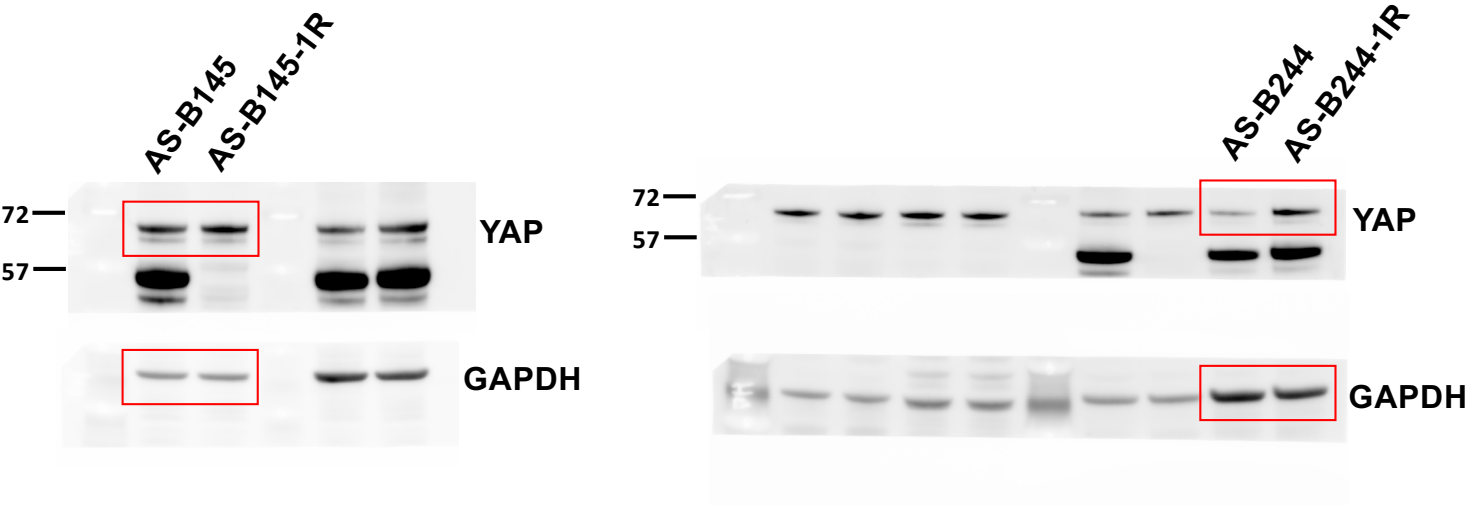

b.

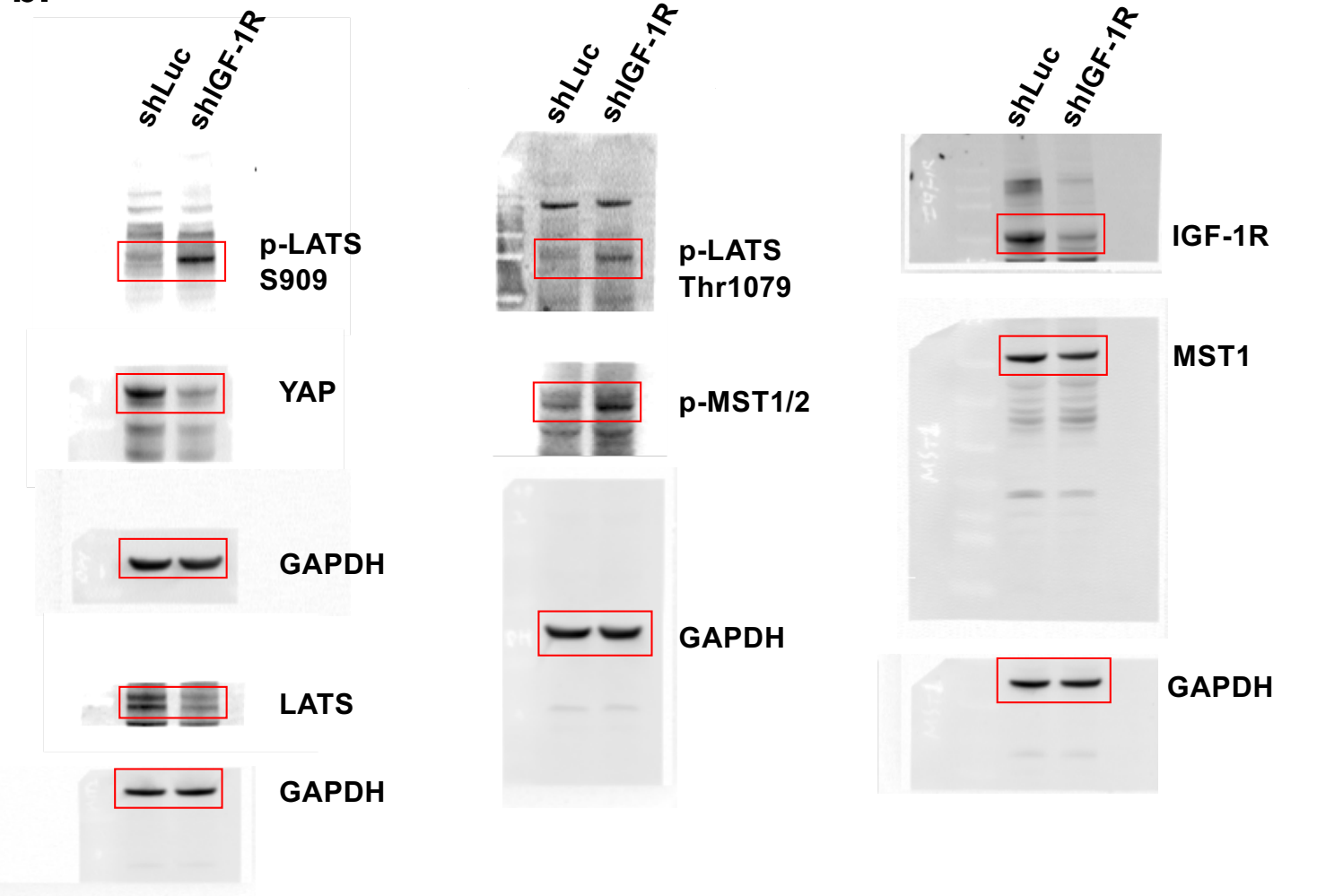

C.

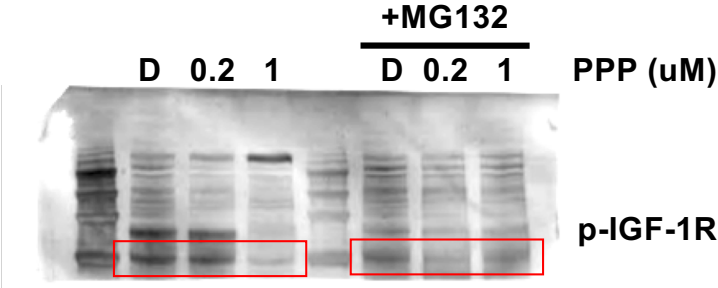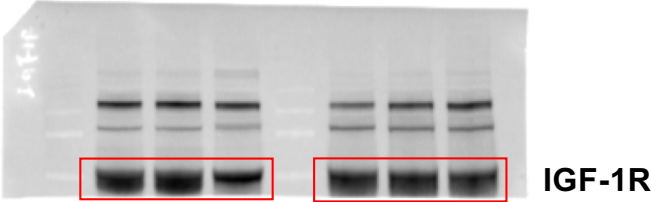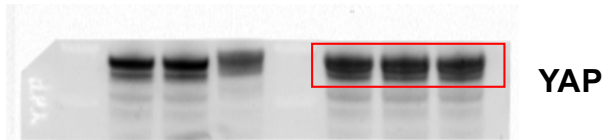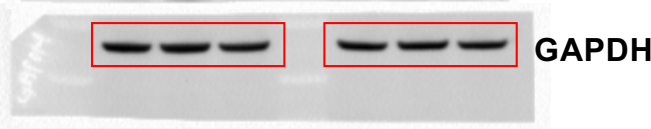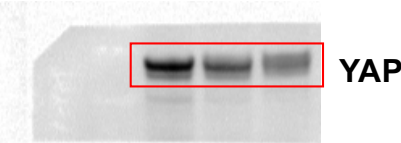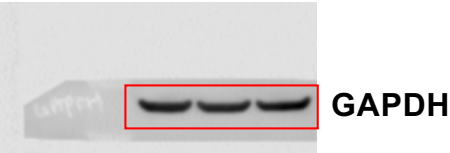

d.

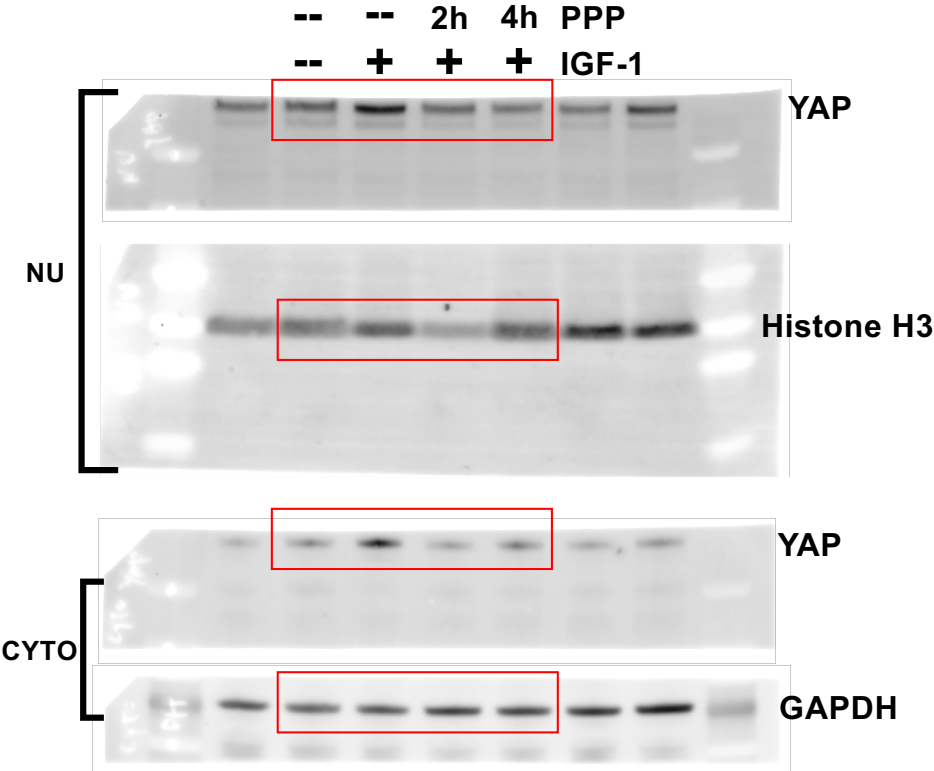

Supp. Figure 2

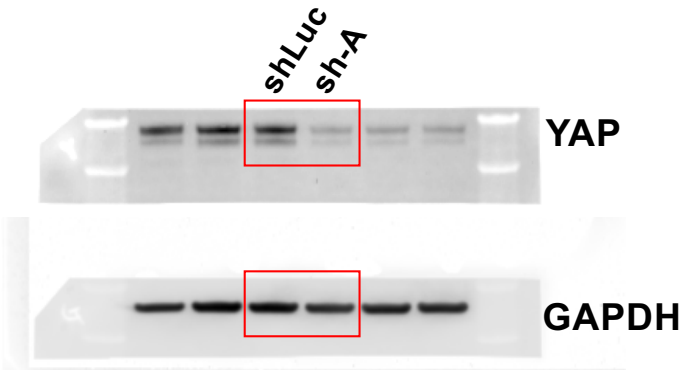

Supp. Figure 3

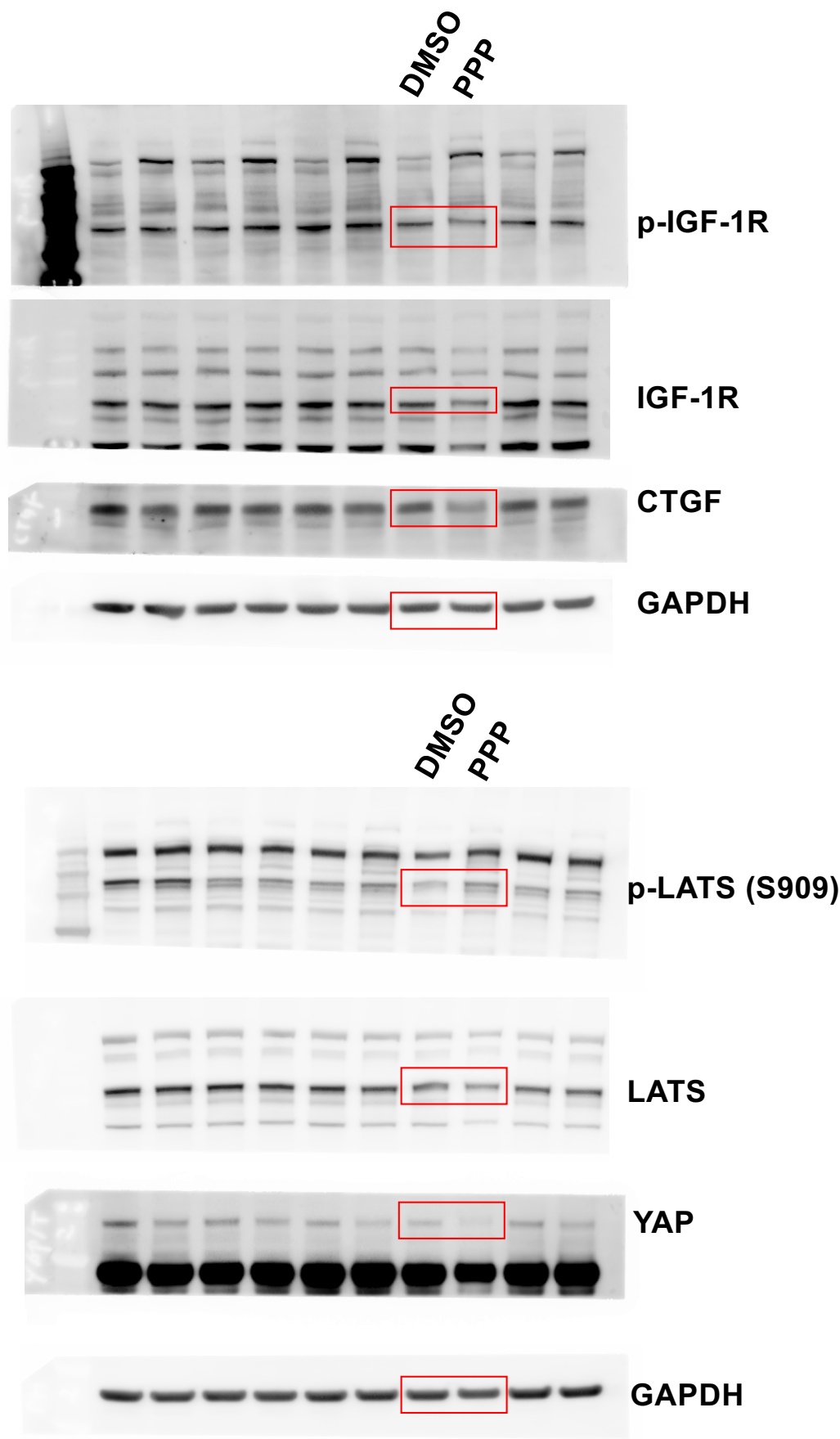

Supp. Figure 4

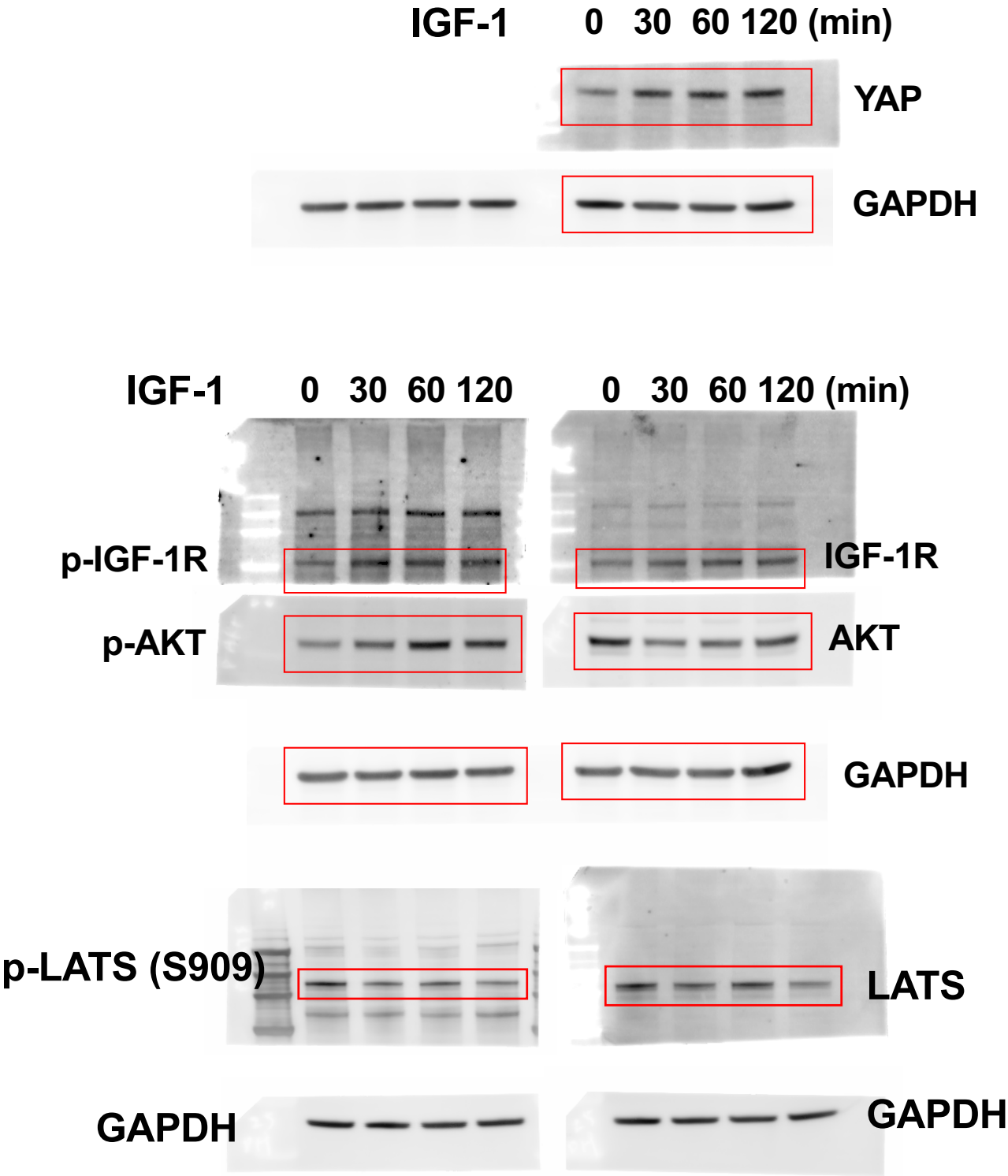

Supplement: Supplementary file 4 — Additional file 3. Uncropped western blots for Figs. 1, 3, and Figs. S2–4 in Additional file 1 [file 12964_2023_1088_MOESM4_ESM.pdf]
